# Building equity from the ground up: a community-academic model in Seward County, Kansas

**DOI:** 10.3389/fpubh.2025.1662968

**Published:** 2025-08-26

**Authors:** Kara Knapp, Clarissa Carrillo Martinez, Sarah Foreman, Susan Lukwago, Julie Foster, Kay Burtzloff, Vicki Collie-Akers, Katherine Atcheson, Sarah Finocchario-Kessler, Christina M. Pacheco

**Affiliations:** ^1^Department of Family Medicine and Community Health, University of Kansas Medical Center, Kansas City, KS, United States; ^2^Department of Population Health, University of Kansas Medical Center, Kansas City, KS, United States; ^3^Liberal Area Coalition for Families, Liberal, KS, United States

**Keywords:** community-academic partnership, health equity, rural health, COVID-19, community health workers, community coalitions, social determinants of health

## Abstract

**Background:**

Academic-community partnerships are vital to addressing health disparities, particularly in rural and diverse communities. This case study highlights a partnership between the Liberal Area Coalition for Families (LACF) and the University of Kansas Medical Center (KUMC) formed through the Communities Organizing to Promote Equity (COPE) initiative. Located in Seward County, Kansas—a region marked by cultural diversity, linguistic complexity, and high social vulnerability—this collaboration leveraged community strengths and academic resources to co-develop and implement equity-driven strategies.

**Methods:**

The partnership established a Local Health Equity Action Team (LHEAT) composed of community members, stakeholders, and public health professionals, supported by a Regional Community Lead (RCL) and Community Health Workers (CHWs). The LHEAT addressed barriers to food access, COVID-19 testing and vaccination, and sustainable public health services through inclusive bilingual engagement models, culturally relevant services, and data-informed planning. Within two years, the LHEAT grew to over 50 members, launched 33 initiatives, and met nearly 90% of 647 identified client health needs.

**Discussion:**

Lessons learned emphasize the importance of beginning with local assets, adapting strategies to context, and building trust over time. The LACF-KUMC partnership showcases how community-led coalitions, supported by responsive academic institutions, can drive upstream systems change. This model underscores the need for flexible, sustained investment in local leadership and participatory evaluation to foster resilience, improve health outcomes, and promote equity across underrepresented communities.

## 1 Introduction

Academic-community partnerships are increasingly recognized as powerful tools for addressing health inequities and the social determinants of health, especially in rural and diverse communities disproportionately impacted by COVID-19 (1–3). These partnerships thrive when built on foundational principles such as mutual trust, shared accountability, and sustained community engagement.

Communities historically marginalized in public health and research—such as American Indian, Hispanic/Latino, and Black/African American populations—have identified mistrust, language barriers, and a lack of cultural relevance as significant challenges (1, 4). In response, effective partnerships prioritize transparency, co-leadership, and respect for lived experience (3, 5). Approaches like Community-Based Participatory Research (CBPR) and collective impact frameworks foster trust by valuing local knowledge, centering community voices, and establishing shared goals (3, 5, 6).

This case study explores one such partnership between the Liberal Area Coalition for Families (LACF) and the University of Kansas Medical Center (KUMC), which emerged to respond to urgent needs during the COVID-19 pandemic and expanded into broader, equity-focused community health initiatives. Through participatory meetings and inclusive strategies, LACF and KUMC co-developed solutions aligned with community-identified priorities. The collaboration illustrates how investing in local leadership, delivering culturally responsive services, and aligning academic resources with community strengths can lead to sustainable, multilevel change in rural Kansas.

### 1.1 Community context and foundational partnerships

#### 1.1.1 Liberal, Kansas

Liberal, Kansas, is the county seat of Seward County, which is located in the state’s southwest corner near the border of Oklahoma. About 19,107 people live in Liberal (7). The area serves as a hub for the agriculture, natural gas, oil, and meatpacking industries, which have drawn a diverse workforce to the region.

As of 2023, 68.6% of the population identified as Hispanic or Latino (of any race). In terms of race, 38.1% identified as White, 25.3% identified as some other race, and 29.1% identified as two or more races. While still relatively small, Liberal, KS has an increasing number of residents who identify as African American or Black (3.1%), American Indian or Alaska Native (1.5%), and Asian (2.6) (7). According to the 2023 ACS 5-Year Estimates (7), 29.1% of Liberal’s population was foreign-born, making it one of the Kansas communities with the highest proportion of foreign-born residents. Additionally, 61.1% of individuals aged 5 and older speak a language other than English at home, and approximately 26 different languages are spoken by families in the USD 480 Liberal School District (8).

Like many rural areas, Liberal, Kansas, faces several daunting health and social challenges. County Health Factors Rankings in Seward County worsened between 2022 and 2023 leaving the county to rank 97 out of 104 counties (9). Limited availability of primary care and low engagement in preventive screenings are likely contributing factors. Additionally, Seward County’s overall CDC/ATSDR Social Vulnerability Index score was 0.9712 in 2022, indicating a high vulnerability level (10). Social vulnerability reflects a community’s ability to prepare for and respond to disasters, whether natural or human caused. The Social Vulnerability Index uses 16 census-based factors, grouped into four thematic areas, to assess vulnerability at the census tract level. The census tracts encompassing Liberal ranked within the top two quartiles of “highest vulnerability” across the four categories: socioeconomic status, racial and ethnic minority status, housing type/transportation, and household characteristics (11).

#### 1.1.2 Liberal area coalition for families (LACF)

Established in 2001, community members created LACF to identify service gaps, pursue grant funding, and develop cross-sector strategies to meet community needs. The initial effort grew into a vibrant coalition, averaging 45 members at each monthly meeting and representing 30 different community organizations/entities. Over the years, LACF has emphasized community-centered, equity-driven practices and expanded its influence to affect local policy and increase access to community-identified services. Amid the COVID-19 pandemic, LACF entered an academic-community partnership with KUMC through the Communities Organizing to Promote Equity (COPE) project, gaining additional resources to deepen and extend its work.

#### 1.1.3 Communities organizing to promote equity (COPE)

The COPE project, active in Seward County from January 1, 2022 to December 31, 2023 previously described in detail elsewhere (12), is a community-academic partnership designed to strengthen the capacity of local, community-driven efforts to address health disparities exacerbated by the COVID-19 pandemic. COPE worked with local communities to establish Local Health Equity Action Teams (LHEATs) across 20 Kansas counties to support community-led strategies addressing social determinants of health. The Regional Community Lead (RCL) played a critical role in bridging academic and community partners within the COPE project. RCLs are community-based leaders hired by the academic partner to support the implementation of the COPE project. Each RCL resides in the region they serve and is selected for their deep knowledge of the local context and strong community relationships. RCLs provide technical assistance to the LHEATs as they identify community priorities and develop strategies to address them. This includes guiding teams in how to effectively leverage their $50,000 project budgets toward sustainable, community-driven solutions to reduce health disparities and ensure local voices remain central to decision-making.

In Seward County, LACF’s coalition served as the LHEAT, which met in-person monthly to discuss community needs and priorities and brainstorm approaches to address those needs. COPE hired a community engaged, bilingual, Liberal resident as the RCL for the state’s Western region who brought deep experience and strong community ties. The LHEAT’s ability to navigate linguistic and cultural barriers was essential to fostering inclusive engagement with both English and non-English speakers.

## 2 Translating partnership into action: Seward County LHEAT in practice

The partnership between LACF and KUMC, supported through the COPE initiative, laid the foundation for actionable, community-driven change in Seward County. By integrating the LHEAT model, the coalition expanded its reach, deepened community engagement, and responded with concrete strategies that addressed local needs, ranging from inclusive coalition-building to sustainable food access and accessible public health services.

### 2.1 Creating a multilingual, inclusive space for community participation

As a group, the LHEAT recognized that early meetings lacked representation from community members with Hispanic identities and did not reflect the full diversity of the community in terms of race/ethnicity, language, and lived experiences. In response, the team made intentional efforts to create a more inclusive and accessible space. Initial strategies included bilingual promotional and communication materials, which successfully increased participation, particularly among Spanish-speaking residents. However, language barriers remained during in-person meetings, as conversations tended to be dominated by either English or Spanish, limiting engagement for monolingual attendees. Observing the impact on participation, the RCL and LHEAT Lead recognized the need for more effective methods to ensure that all participants could contribute to the dialogue, regardless of language background.

In response, the LHEAT gathered participant feedback on several proposed formats, including providing translated materials, but still holding the meeting in a single language, using on-demand interpreters, and organizing breakout sessions with bilingual facilitators. These strategies were ultimately deemed ineffective; participants reported feeling “singled out,” “confused,” or “overwhelmed” by time constraints. In response, the team adopted a fully bilingual meeting model in which the LHEAT facilitator led the group in English, and the RCL immediately translated into Spanish. All attendee comments were translated in real-time, ensuring full participation regardless of language. Detailed agendas and strict time management also guided meetings, and individual follow-ups were offered for unresolved discussion items. Members reported this format fostered greater trust, empowered participation, and strengthened decision-making and cohesion among LHEAT members.

### 2.2 Expanding sustainable food access during and after COVID-19

Through community dialogue and CHW-led outreach, food insecurity emerged as a recurring concern, particularly for families working irregular hours who struggled to access limited pantry hours. These patterns were reinforced by CHW data collected during client intakes and follow-up visits. In response, LACF quickly mobilized resources and coordinated partners to provide emergency food assistance. With support from the LHEAT, these efforts evolved into longer-term strategies to address persistent gaps in food access. Community feedback revealed that local food pantries operated on limited schedules, making it difficult for many families to receive aid. To address this, the LHEAT helped LACF establish a no-barrier, on-call food pantry accessible seven days a week. Within months, the pantry served more than 420 families. Recognizing the importance of cultural relevance and dietary needs, the LHEAT expanded offerings to include culturally appropriate foods and partnered with K-State Research and Extension to deliver free nutrition courses on food preparation using pantry items.

Infrastructure improvements also supported sustainability. The purchase of a commercial freezer, supported by the LHEAT, enabled LACF to increase storage of fresh produce, which was added to distribution boxes. The LHEAT also contributed to the launch of a seasonal Saturday Farmers Market (May–September), further increasing sustainable access to fresh fruits and vegetables in the community.

### 2.3 Bringing COVID-19 services to the community

The LHEAT identified limited access to COVID-19 services through conversations with partners (e.g., local schools and the health department), as well as direct community feedback and CHW-reported barriers. Services were primarily offered by the County’s Emergency Preparedness Team during weekday business hours, which posed a significant challenge for residents working during those times. In response, the LHEAT prioritized community-led service delivery models to expand access and better accommodate residents’ schedules. Drawing on lessons learned from RADx-Up initiative (13), the team launched resource distribution events that provided COVID-19 test kits, cleaning supplies, and hygiene items. These kits were also made available through food pantries and direct delivery upon request.

When similar barriers emerged during the vaccine rollout, the LHEAT collaborated with the local health department to provide Saturday vaccination clinics. These expanded offerings allowed residents more flexibility to access COVID-19 and other routine vaccinations. Importantly, these efforts also generated clear, community-driven evidence of demand for more flexible service hours. The increased turnout and engagement at weekend clinics provided a compelling case for county commissioners, demonstrating the need and public support for expanded operating hours at the local public health department. By bringing services directly to the people and aligning with community preferences, the LHEAT improved access, reduced barriers, and built trust around public health interventions.

### 2.4 LHEAT retention and community impacts

LHEAT leads were identified and trained across all participating counties by Spring 2022. In Seward County, one change occurred in the lead position over the two-year data collection period. In addition to the lead, four community health workers (CHWs) were hired to support the work of the LHEAT, all of whom remained active throughout the project. The Seward County LHEAT began with approximately 15–20 community members and stakeholders in 2021 and expanded to more than 50 members over the course of the project, demonstrating growing community engagement and sustained commitment to equity-focused collaboration. The academic partner collected demographic data from LHEAT members in 2022 and 2024 to assess coalition make-up and representation. These data are presented in Table 1.

**Table 1:** Demographic characteristics of Seward County LHEAT members in 2022 and 2024.

Demographic category	2022 Data *N* = 30		2024 Data *N* = 25	
	*n*	%	*n*	%
Role on LHEAT^a^				
Paid Staff (LHEAT Lead and CHWs)	5	16.7%	5	20.0%
Community members	9	30.0%	11	44.0%
Organizational representatives	12	40.0%	8	32.0%
Other	4	13.3%	3	12.0%
Gender				
Female	28	93.3	23	92.0%
Male	1	3.3%	2	8.0%
Non-disclosed	1	3.3%	0	0.0%
Racial and ethnic diversity^a^				
White	12	40.0%	10	40.0%
Hispanic/Latino	14	46.7%	13	52.0%
American Indian or Alaska Native	1	3.3%	0	0.0%
Asian	1	3.3%	0	0.0%
African American or Black	2	6.7%	3	12.0%
Locations live(d) ^a^				
Rural	26	86.7%	21	84.0%
Urban	4	13.3%	7	28.0%
No stable place to live	0	0.0%	2	8.0%
Experiences at the table^a^				
Immigrants	9	30.0%	5	20.0%
Adult child of immigrant(s)	9	30.0%	7	28.0%
Older Adult (65+)	4	13.3%	4	16.0%
Economic hardship	1	3.3%	2	8.0%
Unhoused	2	6.7%	1	4.0%
LGBTQQIA2S	2	6.7%	3	12.0%
Experienced disabilities	0	0.0%	2	8.0%
Religious minority group	0	0.0%	1	4.0%
English language learner			2	8.0%

^a^Questions related to LHEAT roles, racial and ethnic diversity, locations lived, and experiences at the table were “select all that apply”; therefore, frequencies and percentages may not add to 100%.

Demographic data collected in 2022 (*n* = 30, ~100% response rate) and 2024 (*n* = 25, ~50% response rate) suggest that the Seward County LHEAT has grown more diverse and reflective of the local community. Community members made up an increasing share of the LHEAT, rising from 30% in 2022 to 44% in 2024, indicating deeper grassroots engagement. Across both years, the LHEAT remained predominantly female (over 90%) and showed modest gains in racial and ethnic diversity, with Hispanic/Latino representation growing from 47 to 52%, and Black or African American participation increasing from 7 to 12%. Members also reported lived experiences relevant to the coalition’s equity-focused mission, including immigrant or first-generation backgrounds, housing instability, disability, and LGBTQIA2S + identities. While the lower response rate in 2024 may limit generalizability, the data overall reflect ongoing efforts to build a coalition that mirrors the diverse populations it serves.

Seward County’s LHEAT participated in 33 initiatives and activities (e.g., new practices/procedures, new or modified programs, new or modified policies) during the project period. Of these, 24% (*n* = 8) focused on food accessibility, with the newly established food pantry serving approximately 420 families in its initial months. Another 21% (*n* = 7) of activities addressed COVID-19 services and vaccinations, reaching an estimated 1,330 individuals. These efforts were supported by 18 community partners from a broader network of 119 partnerships established through the LHEAT. CHWs engaged 299 clients who collectively identified 647 health-related needs. CHWs provided direct assistance or coordinated with partners to address 89% (*n* = 580) of these needs. Among them, 4.5% (*n* = 29) were related to COVID-19 services and 30% (*n* = 195) to food access. Clients were referred to 40 partner organizations, resulting in a 100% resolution rate for COVID-19-related needs and a 90% resolution rate for food-related needs.

The data highlight the breadth of the LHEAT’s impact—from expanding food access and public health services to fostering a more inclusive and engaged coalition. These outcomes were made possible through the strong foundation of the LACF-KUMC partnership and the active involvement of community members, CHWs, and organizational stakeholders. Beyond the numbers, the collaboration also offered valuable insights into what it takes to build and sustain effective community-academic partnerships in rural settings. The following section reflects on key lessons learned from the perspective of the LACF, offering guidance for future efforts to advance equity through community-driven solutions.

## 3 Lessons learned

Staff, partners, and community members reflected on the lessons learned from the collaborative experience. Their distilled lessons learned are described here.

### 3.1 LACF perspective

#### 3.1.1 Doing what makes sense for our community

As longtime residents and collaborators, LACF staff brought deep local knowledge to the partnership. Integrating the academic partner and the LHEAT model introduced new perspectives on leadership, shared decision-making, and inclusive engagement. Previously, priorities were identified and addressed internally, often shaped by competing agendas. Through the LHEAT, LACF adopted a more participatory approach, valuing diverse perspectives and engaging community voices at every stage of the work. Today, LACF emphasizes listening first, building consensus, and implementing plans collaboratively to drive lasting systems change.

#### 3.1.2 Monitoring and evaluation matter

Prior to the COPE partnership, LACF lacked capacity for large-scale data collection and relied primarily on informal community feedback to assess progress. While this provided useful insights, the absence of robust data limited the ability to fully evaluate or refine strategies—particularly in areas like food access and COVID-19 response. Through the academic partnership, LACF gained access to technical assistance and training in both qualitative and quantitative evaluation. KUMC-led evaluation efforts helped identify best practices and inform continuous improvement. LACF now recognizes evaluation as a critical area for growth and is seeking continued collaboration to build this capacity.

#### 3.1.3 Support through challenges builds sustainability

Sustaining momentum in community work often requires navigating institutional differences. LACF initially found the more structured approval processes of the academic partner to be slow and, at times, frustrating. Yet, rather than stall progress, the LHEAT and CHWs used this time to deepen outreach, build relationships, and develop more strategic, upstream solutions. What was once seen as a barrier became an opportunity to align priorities, strengthen the partnership, and foster more sustainable, systems-level change. This experience underscored the importance of trust, patience, and persistence in building enduring collaborations.

### 3.2 Regional community lead perspective

#### 3.2.1 Language as a barrier to belonging

As an immigrant who did not speak English as their first language, the RCL identified language as one of the most significant barriers to community engagement. These language challenges often contributed to intimidating environments and feelings of exclusion. In response, the RCL worked alongside the LHEAT to intentionally create a safe, inclusive space where all voices were heard, valued, and empowered. This environment led to increased participation from community members across various backgrounds, ultimately resulting in broader representation at LHEAT meetings.

#### 3.2.2 Every voice matters

The RCL emphasized the importance of reminding LHEAT members that each person’s lived experience added value to the conversation. This consistent message reinforced that everyone had a role in shaping strategies to meet community needs. By fostering a culture of respect and inclusion, the RCL helped create a space where community members felt empowered to engage meaningfully in decision-making processes.

#### 3.2.3 Patience and small wins lead to sustainable change

Another key lesson was the importance of patience and celebrating small wins. For the RCL and community members, shifting focus from addressing immediate needs to developing long-term, upstream solutions was challenging, as day-to-day struggles remained ever-present. However, by participating in cross-community dialogs through COPE, the RCL was able to learn from other leaders across Kansas and apply new insights to local strategy. The LHEAT’s emphasis on long-term impact and regular recognition of incremental progress helped sustain engagement, build community cohesion, and nurture a shared sense of purpose.

### 3.3 Academic partner perspective KUMC

#### 3.3.1 Start with community strengths

One of the most important lessons from this partnership was the value of recognizing and leveraging the assets already present in the community. Too often, external initiatives focus on perceived deficits rather than acknowledging the deep knowledge, relationships, and capacity that local organizations and residents bring to the table. From the beginning, LACF’s existing infrastructure, trusted relationships, and long-standing community presence positioned the coalition for success. The role of the academic partner was not to lead, but to support, offering resources, technical expertise, and evaluation tools in service of locally driven priorities.

#### 3.3.2 One size does not fit all

The diversity of Kansas communities meant that no single model could be applied uniformly across all counties participating in COPE. Each LHEAT operated within a unique cultural, geographic, and political context. What worked in one place often required thoughtful adaptation—or complete reimagining—elsewhere. In Seward County, integrating bilingual facilitation, lived experience leadership, and tailored service delivery strategies underscored the importance of local adaptation. The academic partner learned to approach the work with humility, flexibility, and a willingness to listen rather than prescribe. In-person visits to Seward County provided KUMC partners with the opportunity to witness the community context firsthand, something that cannot be fully understood from afar. Experiencing the local environment, infrastructure, and daily realities afforded the academic team a better appreciation of the strengths, constraints, and nuances shaping the needs of the community and implementation of the initiatives. This contextual understanding proved invaluable in identifying what strategies may or may not work, and helped ensure that technical assistance and resources were aligned with lived experience.

#### 3.3.3 Partnerships take time and trust

Establishing a true community-academic partnership requires ongoing relationship-building, not just project-based collaboration. Early investments in trust, transparency, and shared decision-making laid the groundwork for deeper cooperation as the project evolved. While academic timelines and institutional processes sometimes created friction, aligning with the community’s pace and priorities ultimately strengthened both the partnership and the outcomes. Given the geographic distance between KUMC and LACF (approximately 400 miles) the academic team recognized that sustained, in-person engagement was essential. KUMC faculty and staff made a concerted effort to visit Seward County on a quarterly basis to meet face-to-face with partners, strengthen relationships, and remain responsive to evolving needs. These visits have continued beyond the COPE funding period and remain a critical component of maintaining trust and momentum.

Additionally, KUMC prioritized creating space for community partners to share their own stories and lead dissemination efforts. Rather than speaking on behalf of the community, the academic team worked to amplify local voices by providing support for abstract development, presentation coaching, and conference logistics. These efforts empowered community members to present their experiences and lessons learned at regional and national venues—including the American Public Health Association’s annual meeting (14, 15). By centering lived experience in dissemination, the partnership affirmed that communities are not just implementers, but also knowledge producers whose insights are essential to advancing equity-focused public health work. The success of the Seward County LHEAT affirmed that when community wisdom is centered and academic resources are applied responsively, meaningful and sustainable change is possible.

## 4 Conclusion

As of 2025, although the COPE initiative’s formal funding has ended, the academic-community partnership between KUMC and LACF remains active and continues to evolve. Building on the foundation established through COPE, the partnership secured new funding through the CDC’s Racial and Ethnic Approaches to Community Health (REACH) initiative. This support has enabled the LHEAT to sustain its work and expand community-led strategies focused on increasing access to fresh fruits and vegetables, promoting physical activity, and supporting breastfeeding among families. The LHEAT continues to meet regularly, and KUMC remains a responsive academic partner providing grant writing support and technical assistance to advance emerging community priorities. These continued efforts reflect a long-term commitment to equity, shared leadership, and systems-level change grounded in local context and community voice.

Academic-community partnerships are most impactful when they build on local strengths, prioritize community-identified needs, and promote shared learning. The partnership between KUMC and the LACF, supported through the COPE project, offers a compelling example of this approach in action. Together, they expanded an existing coalition into a LHEAT that implemented culturally responsive, community-led strategies to address the heightened barriers caused by COVID-19.

Key to the success of the Seward County LHEAT were intentional inclusion, sustained engagement, and trust between partners. By integrating RCLs with strong local ties to anchor the relationship between the community and the academic partner, the collaboration remained grounded in lived experience and responsive to emerging needs. Flexibility, mutual respect, and a commitment to long-term, upstream solutions enabled the LHEAT to grow and thrive in the face of evolving public health challenges.

While rooted in a small, rural community, the Seward County experience offers a promising model for other communities seeking to build equitable partnerships that drive systems change. The model emphasizes adapting to local contexts, centering lived experience, and creating bilingual and inclusive coalition structures. These practices can be applied to other diverse, underserved settings and offer a replicable framework for community organizing, particularly where structural barriers and cultural diversity intersect. The lessons from this collaboration underscore that sustainable change requires long-term investment in community leadership, co-ownership, and trust. As the work continues, future efforts must continue to invest in community leadership, adapt to local contexts, and embed evaluation and reflection as core components of sustainability and impact.

## Data Availability

The raw data supporting the conclusions of this article will be made available by the authors, without undue reservation.
